# Evaluation of the integrated disease surveillance and response system for infectious diseases control in northern Ghana

**DOI:** 10.1186/s12889-015-1397-y

**Published:** 2015-02-04

**Authors:** Martin Nyaaba Adokiya, John Koku Awoonor-Williams, Inuwa Yau Barau, Claudia Beiersmann, Olaf Mueller

**Affiliations:** Institute of Public Health, University of Heidelberg, Im Neuenheimer Feld 324, D-69120 Heidelberg, Germany; Department of Allied Health Sciences, School of Medicine & Health Sciences, University for Development Studies, Tamale, Ghana; Ghana Health Service, Upper East Regional Directorate, Bolgatanga, Ghana; National Primary Health Care Development Agency, Ministry of Health, Abuja, Nigeria

**Keywords:** Integrated, Disease surveillance, Response, Infectious diseases, Data quality, Health information system, Ghana

## Abstract

**Background:**

Well-functioning surveillance systems are crucial for effective disease control programs. The Integrated Disease Surveillance and Response (IDSR) strategy was developed and adopted in 1998 for Africa as a comprehensive public health approach and subsequently, Ghana adopted the IDSR technical guidelines in 2002. Since 2012, the IDSR data is reported through the new District Health Information Management System II (DHIMS2) network. The objective was to evaluate the Integrated Disease Surveillance and Response (IDSR) system in northern Ghana.

**Methods:**

This was an observational study using mixed methods. Weekly and monthly IDSR data on selected infectious diseases were downloaded and analyzed for 2011, 2012 and 2013 (the years before, of and after DHIMS2 implementation) from the DHIMS2 databank for the Upper East Region (UER) and for two districts of UER. In addition, key informant interviews were conducted among local and regional health officers on the functioning of the IDSR.

**Results:**

Clinically diagnosed malaria was the most prevalent disease in UER, with an annual incidence rate close to 1. Around 500 suspected HIV/AIDS cases were reported each year. The highest incidence of cholera and meningitis was reported in 2012 (257 and 392 cases respectively). Three suspected cases of polio and one suspected case of guinea worm were reported in 2013. None of the polio and guinea worm cases and only a fraction of the reported cases of the other diseases were confirmed. A major observation was the large and inconclusive difference in reported cases when comparing weekly and monthly reports. This can be explained by the different reporting practice for the sub-systems. Other challenges were low priority for surveillance, ill-equipped laboratories, rare supervision and missing feedback.

**Conclusions:**

The DHIMS2 has improved the availability of IDSR reports, but the quality of data reported is not sufficient. Particularly the inconsistencies between weekly and monthly data need to be addressed. Moreover, support for and communication within the IDSR system is inadequate and calls for attention.

## Background

The health sector needs timely and reliable information for planning and evaluating interventions [1-3]. This appears particularly important as the world now moves from the era of the Millennium Development Goals (MDGs) to – still undefined – new and more sustainable post-2015 goals, which require timely and accurate data for managers and policy makers to take action [4]. Moreover, the new International Health Regulations (IHR) require WHO member states to strengthen their existing capacity for disease surveillance and response [5]. In sub-Saharan Africa (SSA), disease-specific routine health data of acceptable quality are usually unavailable [6,7]. Data collected through routine reporting from health facilities (Health Information Management Systems, HIMS) are rarely complete and usually not representative, as the poorer but more vulnerable sections of the population do less attend health facilities. Routine reporting is often of limited quality due to several factors such as poor motivation, lack of supervision and inadequate feed-back, and overburdening of staff by multiple disease-specific reporting requests [8]. Besides, community perceptions on specific diseases largely influence their health seeking behaviour and, thus, impact on the representativeness, completeness and quality of facility-based data [9-14]. National survey and population census data are now increasingly available from nearly all SSA countries but do not provide detailed information on diseases while specific studies normally address only a single disease [8].

Disease-specific programs continue to implement their own separate surveillance systems thus leading to a proliferation of indicators and onerous reporting requirements as well as an unacceptable extra administrative burden on health staff [15]. As a potential solution, in 1998, the World Health Organization Regional Office for Africa (WHO-AFRO) initiated a strategy for overall strengthening of disease surveillance in SSA called *Integrated Disease Surveillance and Response* (IDSR) [16,17]. WHO-AFRO played a major role in producing the first version of the IDSR technical guidelines for adoption and modification according to the epidemiological priorities of member states [18]. The primary goals of the IDSR strategy were the integration of multiple existing vertical surveillance systems and linking surveillance data to public health action [19]. Diseases of national priority vary based on endemicity and the public health systems ability to respond. For example, countries outside the meningitis belt usually excluded this disease from their national priorities [20].

During the 1990s, the health system in Ghana faced major outbreaks of cholera, yellow fever and meningococcal meningitis [21]. As a result, the National Surveillance Unit (NSU) was created in 1998 to coordinate communicable diseases surveillance. In 2002, Ghana adopted and implemented the first IDSR technical guidelines, which has since been revised in 2011 because of challenges in the country’s health, social, economic, environmental and technical environment. In particular, the emergence and re-emergence of diseases resulted in the need to review the public health priorities for surveillance and response [22]. A well-functioning infectious disease surveillance system involves a certain number of core activities such as case detection, confirmation and registration, reporting, data analysis and interpretation, outbreak investigation, dissemination, feedback and response. The health system usually supports disease surveillance activities by providing training, supervision and resources [23]. Again in 2012, the district health information management system (DHIMS) was restructured to the District Health Information Management System II (DHIMS2). This is an internet-based system with the overall goal of reducing the reporting burden in primary health care settings [24] and to improve data quality and reliability. The process of establishing DHIMS2 in Ghana started at the beginning of 2011. Prior to full-scale implementation, the DHIMS2 software was adapted to suit the local needs and context. By the end of February 2012, all training and trials were completed and the system was ready for nation-wide implementation. Since then, the IDSR data is required to be reported through DHIMS2 network. The objective of this study was to evaluate the IDSR functioning and its data quality on the DHIMS2 in northern Ghana.

## Methods

### Study setting

**Figure 1:**
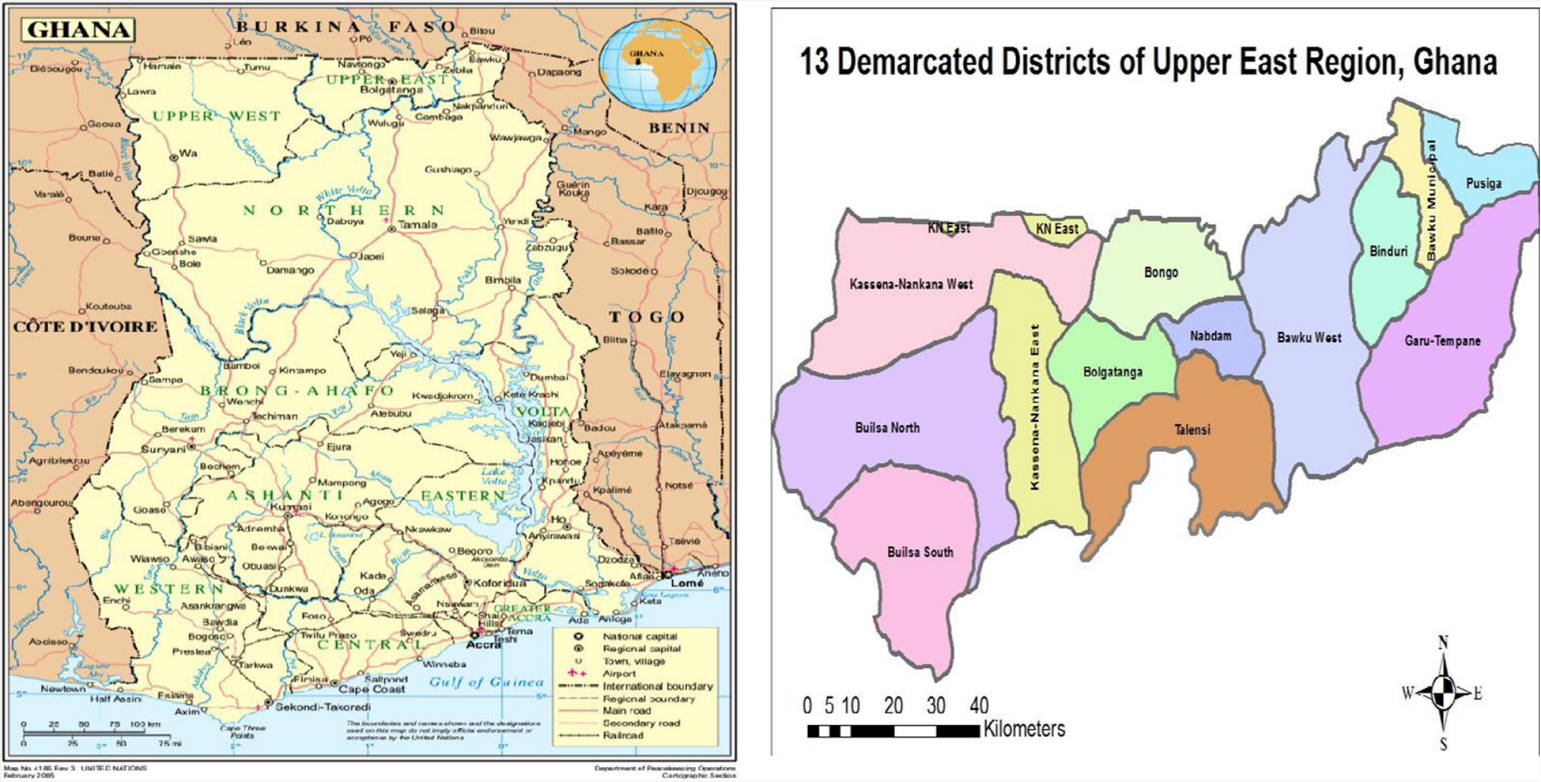
**The study area in northern Ghana.** Source: UN Department of Peacekeeping/Cartographic section and Map from UER Health Direcorate.

Ghana is located in West Africa and composed of 10 administrative regions, which are further divided into 216 districts. Administratively, the health system has four sub-national levels: region, district, sub-district and community. The districts and regions are common for the entire public sector, while the sub-districts are peculiar to the health sector. Kassena Nankana East (KNE) and Kassena Nankana West (KNW) are two of the thirteen districts which constitute Upper East Region (UER) in the northern part of the country [25]. The study covers the entire UER as well as the two districts (Figure 1). The KNE district is further divided into three portions and separated from the KNW district due to the decentralization and political structure of the country. The study area is characterized by Savannah vegetation with a rainy reason from May to September. In 2010, the UER population was 1,046,545 with an annual growth rate of 1.2% according to the 2010 Population & Housing Census [26]. Children of under five years of age in the region constitutes 20% of the total population [27]. The local economy is mainly based on subsistence agriculture. The majority of the people live in rural settings and households are grouped into extended family units or compounds. The compounds are located far from each other yet the people depend largely on communal life style [28].

### IDSR and DHIMS2 functioning

The IDSR is focused on diseases and events of national and international concern. To date, the IDSR system still depends on paper-based data production from the peripheral health facilities. However, DHIMS2 is internet-based and the health system requires disease surveillance data to be transmitted only through the DHIMS2 network from the district, to regional and national levels.

Health information data collection starts with the registers and tally sheets at the health facility level. There are various registers ranging from out-patient, in-patient, consulting room and laboratory registers. At the district and regional hospitals, some of these registers have already been computerized. At the end of each week, month or quarter, summary reports are prepared at the health facility level and submitted to next higher level. Data from health facilities are normally summarized into sub-district reports. A sub-district is described as a health implementation centre/unit within the district which serves a maximum population of 30,000. It provides basic curative care, prevention, maternity and primary health care services [22]. From the sub-district, the health facility reports and its own reports are sent to the District Health Directorate (DHD). At the DHD, data from the paper-based forms are entered into the DHIMS2 network by district health information officers. Data sent from the DHD to the regional health directorate on the DHIMS2 network are merged into a regional database. At the regional level, changes to the data submitted by the districts are not possible. Therefore, if inconsistencies are discovered, corrections are made at the DHD after consultations with the specific health facility and the data re-sent to the region. From the region, the data is then sent to the Centre for Health Information Management (CHIM) office at the national level through the DHIMS2 network.

The standard tally sheet is used daily at each health facility to keep an accurate account of the various priority diseases and events. This is supposed to be conducted at the end of every clinic session and at the end of every week. The sum of the daily standard tally data at the end of the week constitutes the weekly IDSR report for the specific health facility. At the end of the month, the health information or disease surveillance officer sum up the weekly summaries to form the monthly IDSR report.

For disease surveillance, data is collated into standardized reporting forms at the health facility level (weekly or monthly IDSR forms), based on the registers of the health facilities. Community-based surveillance volunteers were trained in some communities to assist on early disease detection. There are 20 immediate notifiable diseases and another 23 diseases that have to be reported weekly and monthly respectively. In total, 43 diseases and events are reported monthly in the IDSR system [22]. On each disease, information is collected on suspected cases, laboratory confirmed cases, and deaths. Disease information entered into the DHIMS2 network at DHD is automatically available to all electronic users of the system. Although DHIMS2 requires an internet connectivity, it allows for offline data entry in case of unstable connection [29,30]. To ensure confidentiality and security, DHIMS2 users have been assigned unique user names and passwords. There are other reports on the network such as disease-specific routine vaccination coverage and health insurance status [30].

The health facility weekly IDSR data is supposed to be submitted to DHD on the Tuesday of the following week. If one of these reports is not submitted in time, the DHD officer makes a follow-up using mobile phone calls to get the information. Health facility officers can also “submit” the weekly reports orally or by text message through their handy to the district officer. The districts then collate all the health facility weekly reports and submit them on the DHIMS2 network on the following Thursday. If the IDSR summary reports are not submitted on the expected date, the regional officers also make phone calls to the district officers for the expected report to be entered into the DHIMS2 network. The monthly IDSR data from the health facilities are reported to the district on 5^th^ day of the following month. The district then enters the monthly reports into the DHIMS2 network before 15^th^ of the following month. The district and regional health information and disease surveillance officers are mandated to conduct regular supervisory visits to verify, validate and enter data into DHIMS2. This aspect is supposed to ensure that missing data, delayed reports and errors are controlled. Once the district IDSR reports are entered into the DHIMS2 network and validated (through supervisory visits at the district offices and meetings), the information becomes available to users in the health system. Ideally, the sum of the weekly summaries at the end of the month should be the same as the respective figures in the monthly report for the same disease.

### Study design

This was an observational study which employed mixed methods to evaluate IDSR data at the sub-national level. The employed mixed method was the convergent parallel strategy where both quantitative and qualitative data collection took place concurrently. The quantitative and qualitative datasets are given equal weight in terms of analysis and comparison [31]. The Upper East Region is one of three northern regions (Northern, Upper East and Upper West) which received technical and financial support in 2002 for training and supervision to improve IDSR performance [32]. Upper East region was chosen because of its remoteness and thus higher likelihood of infectious disease epidemics occurrence and surveillance problems. KNE and KNW were created from the then Kassena Nankana District (KND) in 2008 and were chosen for convenience to investigate the surveillance practice in more details. The fieldwork took place between July and November 2013.

### Study procedure and data collection

The IDSR data for diseases considered representative of the surveillance system were recorded and analyzed constituting the quantitative part of the study. Malaria, HIV/AIDS, tuberculosis and pneumonia are representing high burden diseases (pneumonia only in children under five years); cholera and meningitis are representing epidemic prone diseases; poliomyelitis and guinea worm are representing diseases targeted for elimination/eradication. Among the selected diseases, the epidemic prone and elimination/eradication targeted diseases are reported both weekly and monthly while the high burden diseases are only reported monthly. Tuberculosis was among the selected diseases but was not analyzed as only quarterly reports were available on the DHIMS2. A total of 36 monthly reports (January 2011 to December 2013) - covering the period before, of and after the DHIMS2 system implementation - were downloaded from the DHIMS2 network for UER, KNE and KNW respectively. In addition, 104 weekly (January 2012 to December 2013) IDSR reports - covering the year of and the year after DHIMS2 implementation - were downloaded from the DHIMS2 network for UER, KNE and KNW respectively. Due to technical reasons during DHIMS2 implementation, weekly IDSR reports were only available for 2012 and 2013. Two research assistants were trained and assisted in the data collection for this study.

The qualitative component of the study centered on individual interviews with six key informants selected from the UER health system. These six individual are routinely involved in the core and support functions of the IDSR and DHIMS2 systems and they have acquired adequate knowledge and experience on functioning at the sub-national levels of the health system. At the DHD of KNE/KNW and at the regional office, two informants representing the disease surveillance and health information units were interviewed. The informants were purposefully selected based on their experience and involvement in either disease surveillance or health information systems functioning. Eligibility was limited to the informants working with the health system for more than a year. However, three of the key informants interviewed were either new at their current positions or they were just about to leave the districts for further education or other districts. A semi-structured questionnaire which focused on the core and support functions of the IDSR strategy was administered to the informants.

### Data analysis

The quantitative data was entered, cleaned and analysed using Stata12. Frequencies, percentages and incidence rates were produced from the analysis representing the selected infectious diseases. The qualitative data analysis began by transcribing and reading all the transcripts multiple times to identify critical responses. The specific themes (based on the IDSR technical guidelines) reported include case detection, case confirmation, reporting, analysis, epidemic preparedness, feedback, supervision, training and resources. Each informant’s perspective on the success and challenges of the IDSR strategy were also captured.

### Ethical considerations

The study protocol was reviewed and approved by the Institutional Review Board of Navrongo Health Research Centre (**NHRCIRB155)** and the Ethics Commission of the Heidelberg Medical Faculty (**S-215/2013**). Permission was also received from the Regional Health Directorate. The qualitative interviews were only conducted after an informed consent was given.

## Results

### Quantitative data

Table 1 shows IDSR monthly data over the three studied years. The incidence of clinically diagnosed (suspected) malaria cases slightly increased from 2011 to 2012 and reached nearly 1 per year for 2012 and 2013 in UER. While in 2011 and 2012, close to one third of suspected malaria cases were reported as confirmed, this proportion increased to nearly half in 2013. The combined population of KNE and KNW represents roughly 20% of the UER population; the number of reported malaria cases is only about 10%.

**Table 1:** **Annual incidence rate of selected infectious diseases in Upper East Region and two Districts from January 2011 to December 2013, Ghana**

	**Kassena Nankana East (KNE)**			**Kassena Nankana West (KNW)**			**Upper East Region (UER)**		
**Year**	2011	2012	2013	2011	2012	2013	2011	2012	2013
**Total Population**	111,263	112,598	113,950	71,515	72,373	73,242	1,059,104	1,071,811	1,084,676
**Malaria**									
Suspected malaria	68,162	51,789	54,079	52,702	68,119	50,869	753,414	937,991	928,273
Confirmed malaria	11,190	12,326	19,266	16,661	15,982	20,981	230,262	322,151	410,731
IR of suspected malaria	0.6126	0.4599	0.4746	0.7369	0.9412	0.6945	0.7114	0.8751	0.8558
IR of confirmed malaria	0.1006	0.1095	0.1691	0.2330	0.2208	0.2865	0.2174	0.3006	0.3787
**Cholera**									
Suspected cholera	1	51	0	0	143	0	18	257	23
Confirmed cholera	0	0	0	0	0	0	0	0	0
IR of suspected cholera /10000	0.0899	4.5294	0.0000	0.0000	19.7587	0.0000	0.1700	2.3978	0.2120
**Meningitis**									
Suspected meningitis	67	68	0	2	10	1	156	392	6
Confirmed meningitis	0	0	4	0	0	0	0	34	15
IR of suspected meningitis /10000	6.0218	6.0392	0.0000	0.2797	1.3817	0.1365	1.4729	3.6574	0.0553
IR of confirmed meningitis /10000	0.0000	0.0000	0.3510	0.0000	0.0000	0.0000	0.0000	0.3172	0.1383
**HIV/AIDS**									
Suspected HIV/AIDS	5	0	1	29	17	9	576	376	421
Confirmed HIV/AIDS	0	0	1	0	1	0	0	61	231
IR of suspected HIV/AIDS /10000	0.4494	0.0000	0.0878	4.0551	2.3489	1.2288	5.4386	3.5081	3.8813
IR of confirmed HIV/AIDS/10000	0.0000	0.0000	0.0878	0.0000	0.1382	0.0000	0.0000	0.5691	2.1297
**Poliomyelitis**									
Suspected poliomyelitis	0	0	0	0	0	0	0	0	3
Confirmed poliomyelitis	0	0	0	0	0	0	0	0	0
**Guinea worm**									
Suspected guinea worm	0	0	0	0	0	0	0	0	0
Confirmed guinea worm	0	0	0	0	0	0	0	0	0
**Under-five population**	22,253	22,520	22,790	14,303	14,475	14,648	211,821	214,362	216,935
**Pneumonia in under-five children**									
Suspected pneumonia*	200	177	377	140	134	284	3,592	3,956	4,954
IR of suspected pneumonia	0.0090	0.0079	0.0165	0.0098	0.0093	0.0194	0.0170	0.0185	0.0228

*Pneumonia cases were only reported for under-five children; no confirmation data was available for suspected cases on DHIMS2.

With regard to suspected cholera cases, there was a clear epidemic (257 cases in UER) in the year 2012, but none of these cases was reported as confirmed. The number of suspected meningitis cases was highest in the first two years of the study period, reaching 392 in the year 2012, but went down to only six in the year 2013. None of the 156 suspected meningitis cases in 2011 was reported as confirmed, while 10% of the cases in 2012 were reported to be confirmed. Surprisingly for the year 2013, six suspected and 15 confirmed meningitis cases were reported for UER. This pattern was also seen in KNE.

For newly reported HIV/AIDS cases, the annual number ranged from 376 to 576 in UER over the study period. The total number of confirmed cases increased from zero in 2011 to 231 in 2013, but such an increase was not seen in the two districts studied. From 2011 to 2013, there were three suspected polio cases reported in UER and no case of guinea worm. The three polio cases, which were reported in 2013, were not confirmed. Suspected pneumonia cases in children under five years of age showed an increasing trend in UER, reaching roughly 5,000 in the year 2013. The surveillance system provides no information on confirmed pneumonia cases.

Table 2 provides the IDSR data and is limited to only four of the selected diseases, which are reported both weekly and monthly. Overall, the number of reported cases appear to be systematically lower in the year 2013 compared to 2012. However, there are large and unexplained discrepancies between these two data sources. Sometimes, the weekly figures are higher than the monthly figures or vice versa. Moreover, confirmed disease reports appeared to occur more frequently in the weekly compared to the monthly reports. Finally, there was one guinea worm case which occurred in the weekly report of 2013, but which was not seen in the corresponding monthly report, and which was also not confirmed.

**Table 2:** **Discrepancies in IDSR data on selected infectious diseases in Upper East Region and two Districts from January 2012 to December 2013, Ghana**

	**2012**		**2013**	
	**Weekly**	**Monthly**	**Weekly**	**Monthly**
**Kassena Nankana East (KNE)**				
Suspected meningitis	135	68	0	0
Confirmed meningitis	43	0	0	4
Suspected cholera	42	51	0	0
Confirmed cholera	5	0	3	0
Suspected guinea worm	0	0	0	0
Confirmed guinea worm	0	0	0	0
Suspected poliomyelitis	0	0	0	0
Confirmed poliomyelitis	0	0	0	0
**Kassena Nankana West (KNW)**				
Suspected meningitis	5	10	2	1
Confirmed meningitis	2	0	0	0
Suspected cholera	60	143	0	0
Confirmed cholera	4	0	0	0
Suspected guinea worm	0	0	1	0
Confirmed guinea worm	0	0	0	0
Suspected poliomyelitis	0	0	0	0
Confirmed poliomyelitis	0	0	0	0
**Upper East Region (UER)**				
Suspected meningitis	397	392	88	6
Confirmed meningitis	141	34	24	15
Suspected cholera	102	257	2	23
Confirmed cholera	9	0	3	0
Suspected guinea worm	0	0	7	0
Confirmed guinea worm	0	0	0	0
Suspected poliomyelitis	0	0	0	3
Confirmed poliomyelitis	0	0	0	0

### Qualitative data

The six key informants were all males. In spite of the different divisions among the informants, their general view on the functioning and quality of the IDSR system was rather similar. On the positive side, the informants agreed that the DHIMS2 has made IDSR reports submission easier and the system is relevant for disease surveillance. On the negative side, inadequate staff, limited resources, ill-equipped laboratories and delays in laboratory confirmation of suspected cases were frequently stated.

### Case detection and confirmation

The standard case definitions for disease surveillance were available in English language at the district and regional offices. The respondents complained about the weak laboratory diagnostic capabilities and inadequate staff at health facilities. In particular newly created districts were reported to often have inadequate resources and poor infrastructure, which affected effective case detection and confirmation.

“*The surveillance objectives are not being met because the staff is inadequate to do the work. Some of the workers lack the “technical-know how” to take specimens for investigation and confirmation. Even where the health worker is good, there are no specimen containers to take the samples. No district hospital exists to handle the specimen in this district. We don’t have resources for surveillance activities”.* Surveillance Unit, **Informant #1**

### Data reporting

The informants stated that priority diseases were regularly reported, but that there were some organizational and technical problems. The districts mainly received paper-based IDSR summary data from the health facilities, but sometimes also electronic reports or phone-based information. This leads to some confusion at the district level and to subsequent inaccuracies with regards to the electronic reports forwarded to the regional office. While easy access to multiple information and improved timeliness of communication was perceived as a positive aspect of DHIMS2, there was uncertainty at the peripheral level as to which channel of reporting is the most appropriate; this results frequently in confusion and missing data. Finally, the fact that certain diseases treated at the health facilities get better reimbursement through the national health insurance scheme leads to potential ‘fake’ reporting of diseases.

“*For me, I think the DHIMS2 has affected the preparation and submission of paper-based IDSR reports in the district. Sometimes, the network fails us when we want to submit the IDSR reports. In my place, when the sub-district delays in submitting their reports, it affects the time I also enter my reports in the DHIMS2. The big people* [senior managers of the health system] *don’t know that the workload is huge. The option of people texting surveillance data has made the sub-districts staff reluctant to complete hardcopies for the districts especially the weekly IDSR reports*”. Information unit, **Informant #2**

*“Sometimes, the case definitions for suspected cases are not properly followed due to claim challenges from the National Health Insurance Scheme (NHIS). There are a series of diseases which are covered by the NHIS. It is easier for the health facilities to access claims with some specific diseases than others. This has necessitated health facilities to register or document diseases which can easily be reimbursed by the NHIS”.* Surveillance unit, **Informant #5**

### Data analysis

The respondents were of the opinion that DHMIS2 has overall improved surveillance of diseases. However, due to the different reporting channels it has become more difficult to validate the given information.

“*The current system is very sensitive. In the region, disease surveillance information dissemination has improved through DHMIS2. Our new DHIMS2 has also made disease surveillance analysis to improve in the region. However, it is now more difficult to conduct data verification due to DHIMS2 network”.* [Due to incomplete paper-based IDSR reports availability for manual verification]*.* Information unit, **Informant # 6**

### Epidemic preparedness

The informants indicated that the availability of emergency stocks of drugs and other essential supplies were inadequate in the districts. The challenges were partly attributed to the district assemblies’ failure to provide budgetary and logistics support as required in the decentralization concept.

*“We all know that there are no stocks and drugs for emergencies in the district. All the essential medicines in the district are used up. Anytime there is an outbreak, we get supplies from the regional health administration. Our district assembly does not honor its obligation to provide emergency stocks of drugs for epidemics”.* Surveillance unit, **Informant #3**

### Surveillance feedback

The informants reported that no real feedback to the periphery does exist (e.g. written report or bulletin). Exceptionally, IDSR data is compiled at the regional level in the form of excel spreadsheet and sent back to the district officers but not to the health facilities. Feedback to the health facilities was only communicated either during the unit head meetings or at the half-year review meetings.

“*In our district, feedback is given during half-year review meetings where peripheral health workers are invited to the district health administration to clarify issues based on the discrepancies detected in their IDSR reports submitted”.* Information unit, **Informant # 4**

### Surveillance supervision

According to the respondents, supervision on disease surveillance is irregular in the region. The common reasons given for the irregular or absent supervisory visits were lack of transport, unplanned meetings at the regional level, inadequate personnel and lack of interest for surveillance activities.

*“I saw the integrated monitoring team once from Accra this year. They [*team from national level] *only come to the region when meningitis reaches its threshold and then you will also see the big organizations (WHO, UNICEF, Ghana Health Service etc.) in the region making supervision”. Surveillance unit,***Informant # 5**

### Training

The informants themselves had received some training on IDSR activities and DHIMS2. Only one of the informants interviewed had a university degree. They were all trained through a surveillance course at the Kintampo College of Health Sciences. In addition, the informants had received some training on surveillance organized by different organizations including WHO and Ghana Health Service. However, they rarely conducted training for other staff members of the health facilities in the district or region. Reasons for not training other staff included lack of funding, unwillingness of heads of facilities to send staff for such training, and missing the opportunity for further information dissemination.

*“We do not have the money for feeding all health facility staff during training. When we are going to organize training we only invite few people to participate and only the heads of health facilities to attend. Unfortunately, they do not disseminate the information they had received when they return to their health facilities*”. Information Unit, **Informant #4**

### Resources

The informants reported that financial, human and material resources were still inadequate for surveillance activities. Also frequent transfer and turnover of the staff had negative effects on the IDSR and DHIMS2 programs.

*“They gave us some white motorbikes and now, not a single one is working. How can we do active surveillance when there are no motorbikes? The staff is inadequate and the clinicians do not cooperate on active disease surveillance. Even the community-based surveillance volunteers lack capacity building but there are no resources. Many donors are not interested in IDSR activities. That is why we don’t have resources”. Information unit,***Informant #2**

## Discussion

This study addresses an important public health topic – IDSR in the frame of a national HIMS. The main finding from this evaluation of the Ghana IDSR System for Infectious Diseases Control is that there are still major challenges to the functioning of this system. Such challenges range from problems with the validity and quality of the data entered into the electronic data bank, to problems with regard to confirmation practices of specific diseases, to problems with funding of surveillance activities, and to problems with supervision and support of the responsible health workers in the periphery of the health system. These findings support similar findings from SSA countries on this topic [20,23,33].

Some studies have reported slight improvements in disease reporting associated with either IDSR or DHIMS2 or both systems [20,24,33,34]. This is not really visible in this study from northern Ghana. Often there are also reports of organizational inadequacies for effective and efficient implementation of disease surveillance [35]. In principle, the new electronic system has the potential to enhance data analysis. However, the utilization of the findings for planning and decision making at the sub-national level was still inadequate in this study conducted in a remote rural African setting. Moreover, improved surveillance data availability may create other problems in relation to data and information management [20]. In principle, the potential for error introduction during data transmission from one level to another using paper-based records should be reduced by internet-based networks [36]. The health staff interviewed in this study has confirmed such a principle benefit of the electronic surveillance procedures. However, in reality the system appears to be still in its infancy and will need major attention and support to reach its potential. In particular, policy makers have to allocate sufficient resources to the neglected field of data availability and quality [37].

Malaria has been reported as the most prevalent disease in the study area, and there was even a slight increase over time. In recent years and with increasing roll-out of effective malaria control interventions in SSA, there was also a change in policy towards treating malaria only after laboratory confirmation [38,39]. This is reflected also in this study by an increased proportion of malaria cases which were reported as confirmed. The proportion of confirmed cases may even be higher, as it was reported by the interviewees that they did not always had sufficient access to the consulting and laboratory registers.

Pneumonia remains a major infectious disease, affecting both children and adults. However, only pneumonia in under-five children is considered a public health priority in Ghana and thus required to be reported in the IDSR system. There has also been an increase in the number of reported pediatric pneumonia cases during the study period in northern Ghana. As in the case of malaria, this could be a true increase due to unknown factors or it could be attributed to improved reporting practice. Moreover, the clinical diagnosis of malaria and pneumonia largely overlaps in malaria-endemic regions [38].

The number of newly reported HIV/AIDS cases was comparatively small. The country reported already a 66% reduction in new infections since 2012 [40]. However, given the chronic nature of HIV/AIDS it is a challenge to differentiate whether the cases were really new cases or cases already reported in former years. It is encouraging that the proportion of confirmed HIV/AIDS cases was sharply increasing during the study period.

Cholera and meningitis are known to occur regularly as epidemics. In this study, the data document a cholera and meningitis epidemic in the year 2012, which is supported by other national reports [27,41]. Ghana belongs to the African meningitis belt. However, recent advances in vaccination provision will likely have an important impact on meningitis figures in the region [41,42]. The finding from this study of very low number of reported cases in the year 2013 may already be attributed to the national campaign of vaccinating children and young adults aged between 1 and 29 years in 2012 with the new meningococcal A conjugate (MenAfriVac) vaccine in the three northern regions [42]. There was no clear and immediate explanation for the inconsistencies between the reported suspected cases compared to relatively more confirmed cases of meningitis in 2013. Upon probing, the managers explained that it was a typographical mistake. They explained that the fifteen (15) cases are rather suspected while the six (6) cases represented the confirmed. However, this observation supports the impression of existing weaknesses in the IDSR system.

Polio and guinea worm disease are both targeted for global eradication [43]. It is reassuring that of the three cases of polio and one case of guinea worm reported in the study area in 2013, none was confirmed.

The wide discrepancies between the weekly and monthly IDSR data on the same disease are a real problem. Largely, it revealed challenges regarding accuracy, reliability and soundness of the IDSR data and may lead to low utilization of health system data for planning and decision-making [24]. Discrepancies are likely caused by a mix of factors including negative attitudes of health workers towards surveillance and lack of commitment to the IDSR activities, as also seen in other SSA countries [44]. The perception that IDSR does not contribute to income generation at the health facilities is a particular problem which needs attention. There were also some problems in the DHIMS2 design. For instance, when zero is entered into the system, the network is unable to display the figure as zero whenever the reports were downloaded. The outcome leads to uncertainty whether the data was actually missing or a zero was entered.

The study has strengths and limitations. It was conducted in a limited geographical area, at the initial stages of DHIMS2 implementation, and the study districts were not randomly selected. Thus the findings are not representative for the whole of Ghana. Nevertheless, the data - which have been derived from different data sources - clearly show major problems of the system and will thus be helpful for further improvement.

## Conclusions

In conclusion, the DHIMS2 has in principle improved the availability of IDSR reports, but the quality of data reported is not sufficient. Particularly, the inconsistencies between weekly and monthly data need to be addressed. Moreover, support for and communication within the IDSR system is inadequate and calls for attention.
